# The roadmap of two-dimensional materials toward next-generation image sensor

**DOI:** 10.1093/nsr/nwae431

**Published:** 2024-11-30

**Authors:** Na Zhang, Decai Ouyang, Yuan Li, Tianyou Zhai

**Affiliations:** State Key Laboratory of Materials Processing and Die & Mould Technology, School of Materials Science and Engineering, Huazhong University of Science and Technology, China; State Key Laboratory of Materials Processing and Die & Mould Technology, School of Materials Science and Engineering, Huazhong University of Science and Technology, China; State Key Laboratory of Materials Processing and Die & Mould Technology, School of Materials Science and Engineering, Huazhong University of Science and Technology, China; State Key Laboratory of Materials Processing and Die & Mould Technology, School of Materials Science and Engineering, Huazhong University of Science and Technology, China

## Abstract

This Prospective highlights the advances and challenges of 2D materials regarding the materials preparation, device integration, multifunctional applications, and comments on their potential as transformative candidates for future image sensors.

With the rapid development of visual information technology, image sensors are increasingly required to meet complex demands, including higher sensitivity, wider detection band, high-density integration, flexibility, and intelligent functionality [1]. However, conventional image sensors still suffer from inherent limitations, such as poor photoelectric sensitivity, narrow detection bands, and lack of multifunctionality, which significantly limit their capabilities in cutting-edge applications. Furthermore, traditional materials with three-dimensional structures are insufficient to achieve the level of integration required by next-generation optoelectronic systems. The rigidity and brittleness make most of them unsuitable for portable and flexible devices. This reveals an urgent need for innovative materials that can not only improve the fundamental performance of image sensors but also expand their functional range, enabling new applications that overcome current technological limitations.

Two-dimensional (2D) materials have attracted great research interest due to their unique properties such as high surface-to-volume ratio, strong light absorption, and mechanical flexibility. Compared with traditional silicon-based image sensors, 2D materials exhibit largely enhanced sensitivity (about 5 orders of magnitude higher than silicon) and a broader detection range, attributed to the strong light-matter coupling and abundant energy band structures (Fig. 1a). Besides, the smooth surface without dangling bonds enables high-density integration of 2D materials with other components (Fig. 1b) [2]. And they are suited for large-scale production and well compatible with CMOS technology (Fig. 1c). Further, the excellent mechanical properties and easily-regulated carrier dynamics of 2D materials make them quite promising for wearable flexible optoelectronic devices and future multifunctional applications [3]. In this perspective, the bottlenecks and development trends of 2D materials toward next-generation image sensors are discussed, focusing on material synthesis, high-density integration, and functional applications.

**Figure 1.**
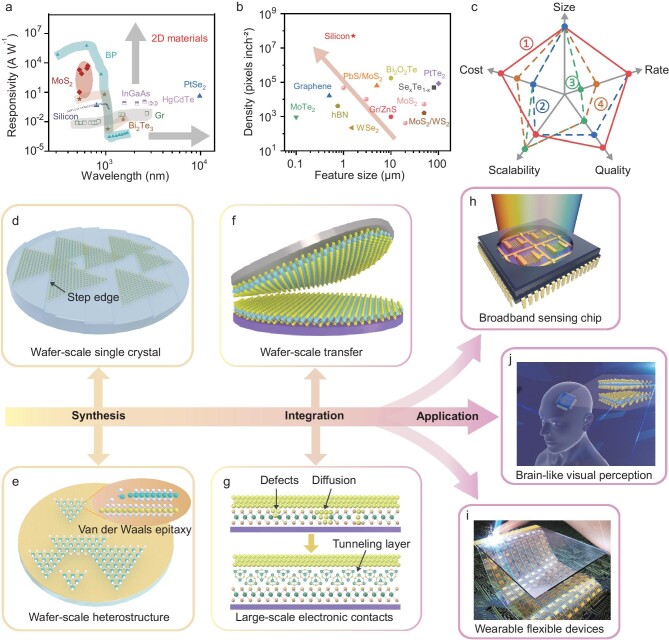
(a) Responsivity versus wavelength of 2D materials and traditional image sensors. (b) Integration density versus device feature size of 2D materials and silicon-based devices. (c) Evaluation of strategies for growing wafer-scale 2D films in terms of film size, growth rate, crystal quality, scalability, and cost. ①: Step edge guide strategy; ②: uniform source supply strategy; ③: metal-organic CVD strategy; ④: chemical conversion strategy. (d–j) Development trends of next-generation image sensors incorporating 2D materials in material synthesis (d, e), high-density integration (f, g) and functional applications (h–j). Reproduced with permission from Ref. [12]. Copyright 2016, Wiley-VCH.

## Material synthesis

The fabrication of wafer-scale 2D single crystal films and their heterostructures lays the foundation for large-scale application of 2D optoelectronic integrated devices. However, the synthesis of wafer-scale 2D single-crystal films faces challenges in ensuring uniformity and crystalline quality. One effective approach for improving the uniformity of wafer-scale films is to control the release of precursors. Metal-organic chemical vapor deposition (CVD) is suitable to grow wafer-scale uniform 2D materials owing to its precise control over precursor ratio, growth time, and rate. Researchers have also achieved the growth of uniform 12-inch MoS_2_ films through an improved source-supply strategy [4]. To obtain 2D single-crystal materials, unidirectional nucleation and seamless stitching offer a reliable strategy by controlling the growth orientation. The step edge guide strategy (Fig. 1d), where nucleation prefers to occur at the step edge and 2D domains grow and merge unidirectionally, is widely considered as a promising technique for growing wafer-scale 2D single-crystals [5].

Besides, reliable methods for preparing wafer-scale 2D heterostructures are still in their infancy. Two-dimensional materials could be stacked arbitrarily through mechanical transfer without being concerned with lattice matching, but the transfer process often introduces interface contaminations and defects. Successive deposition, which involve cycling the deposition step twice, can effectively mitigate these issues. Recently, Gao *et al*. reported a high-to-low temperature strategy and developed a multicycle two-step vapor-deposition process to grow the specific heterostructure films at a wafer scale [6]. It is also critical to control nucleation and growth of the top layer during successive deposition. Seeded growth is to guide the nucleation and growth of the top layer through generating artificial defects or seeds on the underlying material, significantly reducing nucleation density, and improving heterostructure quality [7]. Direct epitaxial growth (Fig. 1e), which involves facilitating lateral van der Waals epitaxy of the top layer and controlling the growth orientation, shows great promise in achieving well-oriented wafer-scale 2D single-crystal heterostructures.

## High-density integration

During the high-density integration process of 2D photoelectric devices, it is indispensable to transfer wafer-scale 2D materials and their heterostructures to various target substrates (Fig. 1f). However, conventional polymer-assisted transfer methods usually introduce cracks, wrinkles, contaminations, and doping, degrading the photoelectric performance of 2D materials. The flexibility of 2D materials makes them susceptible to mechanical deformation during the transfer process. Therefore, continuous support and controlled conformal contact are essential to prevent defects. Moreover, the transfer is primarily governed by differences in surface energy and adhesion force between different layers. Strategies like gradient surface energy modulation and tunable adhesive force tapes are quite promising for obtaining clean, defect-free, ultra-flat films and realizing high-yield transfer [8,9]. Despite recent advancements, most transfer methods remain incompatible with mass production. Thus, the implementation of automated detaching and bonding machines, which allow precise control over the detaching/attaching force, is expected to greatly alleviate these technical issues.

Achieving high-quality contacts between materials and electrodes at the wafer scale remains a key challenge during the high-density integration process. Owing to the atomically thin nature of 2D materials, there exist serious metal/semiconductor interface problems, such as material damage, Fermi level pinning, and unexpected doping. Transferring metal electrodes can avoid the material damage caused by ‘high-energy’ metal deposition and allow the formation of van der Waals interfaces. Introducing a tunneling layer between metal and semiconductor is a reliable approach to effectively mitigating Fermi level pinning. Semimetal–semiconductor contacts can minimize metal-induced gap states and realize superior ohmic contact. However, these methods still face limitations in wafer-scale integration. Employing inorganic molecular crystals with low melting points as tunneling layers offers a promising solution (Fig. 1g). High-quality van der Waals contacts are formed without material damage due to low-temperature sublimation, facilitating large-scale fabrication and device integration [10,11].

## Functional applications

To facilitate the transition of 2D materials from lab to industrial applications, it is essential to explore new functional applications tailored to 2D image sensors. Conventional optoelectronic devices suffer from narrow photodetection bands and poor sensitivity due to their inherent bandgap and limited light absorption. In contrast, 2D optoelectronic devices exhibit superior performance owing to the quantum confinement effect, including ultra-high sensitivity, ultrabroadband detection ability (Fig. 1h), and strong light-matter interaction, which show natural advantages in ultra-weak light imaging, infrared image sensors, and so on.

Additionally, with their superior mechanical properties and adaptability, 2D materials provide distinct advantages for developing ultrasensitive, low power consumption, and multifunctional wearable flexible devices (Fig. 1i) [12]. The direct low-temperature synthesis of 2D materials on flexible substrates is a prerequisite for advancing 2D flexible electronic devices. Strategies like plasma-enhanced CVD or low-temperature precursors have proven to be promising for lowering the synthesis temperature of 2D materials, which is crucial for unlocking their full potential in next-generation flexible optoelectronic devices with enhanced performance and functionality.

Furthermore, the carrier dynamics of 2D materials could be precisely regulated, enabling simulations of biological synapses and neurons at the physical level (Fig. 1j). New image sensors incorporating 2D materials hold great promise for integrating sense, memory, and processing within a single device, thus overcoming the limitations of traditional ‘von Neumann’ architecture [13]. However, early studies on 2D optoelectronic synapse devices have mainly focused on simple and static image recognition. Future development of algorithms tailored for on-chip operations should also be prompted for a dynamic image recognition system to improve the computing power and handle complex tasks.
